# Behavioral experience induces zif268 expression in mature granule cells but suppresses its expression in immature granule cells

**DOI:** 10.3389/fnsys.2015.00118

**Published:** 2015-08-21

**Authors:** Kylie A. Huckleberry, Gary A. Kane, Rita J. Mathis, Sarah G. Cook, Jonathan E. Clutton, Michael R. Drew

**Affiliations:** Department of Neuroscience, Center for Learning and Memory, University of Texas at AustinAustin, TX, USA

**Keywords:** adult neurogenesis, dentate gyrus, hippocampus, immediate-early gene (IEG), memory

## Abstract

Thousands of neurons are born each day in the dentate gyrus (DG), but many of these cells die before reaching maturity. Both death and survival of adult-born neurons are regulated by neuronal activity in the DG. The immediate-early gene (IEG) zif268 appears to be an important mediator of these effects, as its expression can be induced by neural activity and knockout of zif268 impairs survival of adult-born neurons (Richardson et al., 1992; Veyrac et al., 2013). Despite the apparent importance of zif268 for adult neurogenesis, its behavior-induced expression has not been fully characterized in adult-born neurons. Here we characterize behavior-evoked expression of zif268 in mature and newborn dentate granule cells (DGCs). We first quantified zif268 expression in doublecortin-positive (DCX+) immature neurons and in the general granule cell population after brief exposure to a novel environment (NE). In the general granule cell population, zif268 expression peaked 1 h after NE exposure and returned to baseline by 8 h post-exposure. However, in the DCX+ cells, zif268 expression was suppressed relative to home cage for at least 8 h post-exposure. We next asked whether suppression of zif268 in DCX+ immature cells occurs in other behavioral paradigms that recruit the hippocampus. Exposure to Morris water maze (MWM) training, an enriched environment, or a NE caused approximately equal suppression of zif268 expression in DCX+ cells and approximately equal activation of zif268 expression among the general granule cell population. The same behavioral procedures activated zif268 expression in 6-week-old BrdU-labeled adult-born neurons, indicating that zif268 suppression is specific to immature neurons. Finally, we asked whether zif268 suppression varied as a function of age within the DCX+ population, which ranges in age from 0 to approximately 4 weeks. NE exposure had no significant effect on zif268 expression in 2- or 4-week-old BrdU-labeled neurons, but it significantly suppressed zif268 expression in 3-week-old neurons. In summary, behavioral experience transiently activated expression of zif268 in mature granule cells but caused a more long-lasting suppression of zif268 expression in immature, adult-born granule cells. We hypothesize that zif268 suppression inhibits memory-related synaptic plasticity in immature neurons or mediates learning-induced apoptosis of immature adult-born neurons.

## Introduction

The dentate gyrus (DG) is one of the few brain regions that generates neurons in adulthood (Altman and Das, 1965; Cameron and McKay, 2001). Thousands of cells are generated each day in the DG, but many of these die before reaching maturity. The probability of survival is influenced by local neural activity. During the first 2–3 weeks after terminal division, depolarizing extrasynaptic GABA_A_-mediated currents (Ge et al., 2006; Song et al., 2012) support circuit integration and synapse formation (Ge et al., 2006). Between 2 and 3 weeks of age, survival is determined through competitive N-methyl-D-aspartate (NMDA)-dependent signaling (Tashiro et al., 2006). Behavioral manipulations that stimulate neural activity in the hippocampus can increase the survival probability of newborn neurons, presumably through these mechanisms. The intracellular mechanisms through which newborn cell survival is regulated are not well understood, but a recent study implicates the immediate-early gene (IEG) zif268, also known as egr1. Like other IEGs, zif268 expression is rapidly induced by hippocampus-dependent learning, and knockout of zif268 impairs hippocampus-dependent learning (Jones et al., 2001). Zif268 is also expressed in immature adult-born neurons, and newborn neuron survival is impaired in mice lacking a functional zif268 gene (Veyrac et al., 2013). Furthermore, enhancement of newborn neuron survival by hippocampus-dependent learning is not observed in zif268 knockout mice (Veyrac et al., 2013). The data suggest that behavior-induced zif268 expression regulates survival of newborn neurons.

Despite the apparent importance of zif268 expression in neurogenesis, behavior-induced expression of zif268 has not been comprehensively characterized in newborn granule cells. Recent studies characterizing behavior-induced zif268 expression in newborn neurons reported that zif268 expression is very low or undetectable in adult-born neurons less than 3–4 weeks old (Jessberger and Kempermann, 2003; Snyder et al., 2009b; Jungenitz et al., 2014), which is incompatible with the hypothesis that zif268 expression supports the survival of 2–3 week-old neurons. Another study, which identified newborn neurons based on doublecortin (DCX) expression, reported that exposure to the water maze, a hippocampus-dependent spatial memory task, suppressed zif268 in newborn neurons as compared to a home cage condition (Snyder et al., 2012). The authors hypothesized that behavior-induced suppression of zif268 was related to the stressfulness of the water maze procedure. Another possibility, not considered, is that zif268 suppression mediates learning-induced apoptosis of newborn neurons, which has been reported in the water maze (Döbrössy et al., 2003; Dupret et al., 2007). Behavior-induced zif268 suppression in newborn neurons may explain why Snyder et al. (2009a) and Jessberger and Kempermann (2003) failed to detect expression in young neurons: these studies examined zif268 solely in response to behavior or kainic-acid induced seizures and failed to include a home cage condition, which may have revealed stronger zif268 expression in immature neurons.

The current studies addressed several critical questions relating to zif268 expression in newborn neurons. First, we attempted to confirm behavior-induced suppression of zif268 expression in DCX+ immature neurons and to determine whether it occurs in response to behavioral manipulations other than the water maze. Second, we compared home cage and behavior-induced zif268 expression in adult-born neurons of varying ages to determine whether zif268 suppression is general across the DCX+ population or confined to a particular range of cell ages. Our results indicate that multiple behavioral manipulations that induce zif268 expression in mature granule cells simultaneously suppress zif268 expression in newborn neurons. This zif268 suppression is limited to adult-born neurons 2–3 weeks of age. By 6 weeks of age, behavioral experience activates zif268 expression rather than suppressing it. We hypothesize that zif268 expression may be a mechanism through which hippocampus-dependent learning induces apoptosis of newborn neurons too immature to be integrated into memory networks.

## Materials and Methods

### Subjects

Ninety-eight male C57BL/6J mice aged 8–13 weeks were used. Mice were housed in plastic cages with wood chip bedding and maintained on a 12-h light/dark cycle with food and water provided *ad libitum*. Mice in Experiment 1 were housed singly beginning 1 week prior to the experiment; all other mice were group housed (max four mice per cage). To minimize IEG expression associated with transport, mice were moved to the experiment room at least 16 h prior to begin euthanized. Mice were gently handled for 1–2 min per day for at least 3 days prior to experimental procedures. All procedures were approved by the University of Texas at Austin Institutional Animal Care and Use Committee.

### BrdU Injections

Mice were injected with 5′-bromo-2′-deoxyuridine (BrdU; Sigma-Aldrich; 150 mg/kg; 10 mg/ml in 0.9% NaCl) twice per day intraperitoneally for 5 days. Injections occurred 2, 3, 4, or 6 weeks prior to euthanasia (*n* = 11, 12, 12, and 30, respectively).

### Novel and Enriched Environment Exposure

Novel and enriched environment exposure were performed in four gray open field arenas (40 × 40 cm) with opaque walls. The arenas were lighted with white incandescent bulbs (25 lux at the center of the arena). Novel and enriched environments differed in that the latter contained a running wheel, plastic tube, and Lego object, whereas the former did not contain stimuli. Mice were placed in the arena for 10 min or 2 h and were euthanized 2 h after being placed in the arena. Mice receiving a 10-min exposure were returned to the home cage for the interval between arena exposure and euthanasia. Home cage control mice were removed from the home cage and immediately anesthetized for perfusion.

### Morris Water Maze

A circular tank (120 cm diameter) was filled with water, which was made opaque using white tempera paint. The water temperature was maintained at 21°C. An escape platform (8 cm diameter) was submerged 0.5 cm below the surface of the water in the center of one of the four quadrants. The tank was surrounded by black curtains located 30–60 cm from tank walls. Visual cues of varying shapes, sizes and colors were affixed to the curtains. After each trial, mice were placed in a warmed holding cage containing dry paper towels.

Mice were first pre-trained to climb on the platform inside a smaller tank (30 cm diameter). Mice received four pre-training trials per day for 2 days. On each trial, the mouse was placed into the smaller tank and allowed to climb onto the platform. If a mouse failed to climb onto the platform within 1 min, the experimenter guided it onto the platform. Mice remained on the platform for 10 s.

Hidden platform training commenced on the day following completion of pre-training. On each trial the mouse was placed into the maze at one of four start positions, which were spaced equally around the maze and selected randomly on each trial. If the mouse failed to locate the platform within 2 min, the experimenter guided it there. The mouse remained on the platform for 10 s before being removed. The intertrial interval was 4–8 min. Mice received four trials per day for 4 days. The final trial of day 4 was a 60-s probe trial with the platform absent. Mice were video recorded from above, and the swim path was analyzed using Any-maze software (Stoelting, Inc.). Mice were euthanized 2 h after the first trial on day 4 of water maze training.

### Immunohistochemistry

Mice were anesthetized with ketamine/xylazine (150/15 mg/kg) and transcardially perfused with 25 ml of cold 0.1M phosphate-buffered saline (PBS) followed by 15 ml of cold 4% paraformaldehyde (PFA) in PBS. Brains were post-fixed overnight in 4% PFA at 4°C and then cryoprotected in 30% sucrose in PBS at 4°C for at least 2 days. Serial coronal sections (35 μm) were cut through the entire hippocampus on a cryostat and stored in 0.1% sodium azide in PBS.

For DCX and IEG immunohistochemistry, sections were washed in PBS and blocked in 10% normal donkey serum (NDS) in PBS with 0.25% Triton X-100 for 1 h at room temperature. Tissue was incubated with primary antibody in blocking solution overnight (goat anti-DCX 1:1000, Santa Cruz Biotechnology, sc-8066; rabbit anti-egr1 1:1000, Santa Cruz Biotechnology, sc-189). Sections were washed the next day in PBS and incubated with a secondary antibody in blocking solution (Cy3-conjugated donkey anti-goat 1:500, Jackson ImmunoResearch Laboratories, 705–165–147; Alexa Fluor 488-conjugated donkey anti-rabbit 1:500, Jackson ImmunoResearch Laboratories, 711–545–152) for 1–2 h at room temperature. For BrdU immunohistochemistry, sections were denatured in 2N HCl for 30 min at room temperature, equilibrated in 0.1 M Boric Acid (pH 8.0), and then washed in PBS prior to the first blocking treatment. Immunohistochemistry was then performed as described above using primary antibodies against BrdU (rat anti-BrdU clone BU1/75/ICR1, 1:250, GeneTex, GTX26326), NeuN (mouse anti-NeuN clone A60 1:1000, Millipore, MAB377), and zif268/egr1. The secondary antibodies were Cy3-conjugated donkey anti-rat (1:500, Jackson ImmunoResearch, 712–165–153), Alexa Fluor 647-conjugated donkey anti-mouse (1:500, Jackson ImmunoResearch Laboratories, 715–605–150) and Alexa Fluor 488-conjugated donkey anti-rabbit (1:500, Jackson ImmunoResearch Laboratories, 711–545–152).

### Cell Quantification

Zif268+, DCX+, and BrdU+ cells in the granule cell layer (GCL) and subgranular zone (SGZ) were counted by an experimenter blind to the experimental condition. Labeled cells were counted in every 12th section throughout the DG (six sections in total). BrdU+ cells were counted exhaustively under fluorescent illumination (Zeiss Axio Imager M2) using a 40× objective (Plan-Neofluar 0.75 NA). Each BrdU+ cell was examined for co-localization with NeuN and/or zif268. Zif268+ and DCX+ cells were quantified using optical fractionator (Stereo Investigator, MBF Bioscience). Counting was performed using a 40× objective (Plan-Neofluar 0.75NA). The counting frame (approximately 70 × 70 μm) and sampling grids (approximately 150 × 250 μm) were optimized to yield at least 200 counts for each cell type of interest. Each DCX+ cell was examined for colocalization with zif268.

### Statistical Analysis

The counts were analyzed using Graphpad Prism 6 software. All experiments were analyzed using either a *t*-test or a one-way ANOVA, and significant ANOVA main effects were then probed with protected Fisher’s least significant difference (LSD) tests. Alpha was set at 0.05 in all analyses.

## Results

### Opposite Effects of Novel Environment Exposure on Zif268 Expression in Immature and Mature Granule Cells

**Figure 1:**
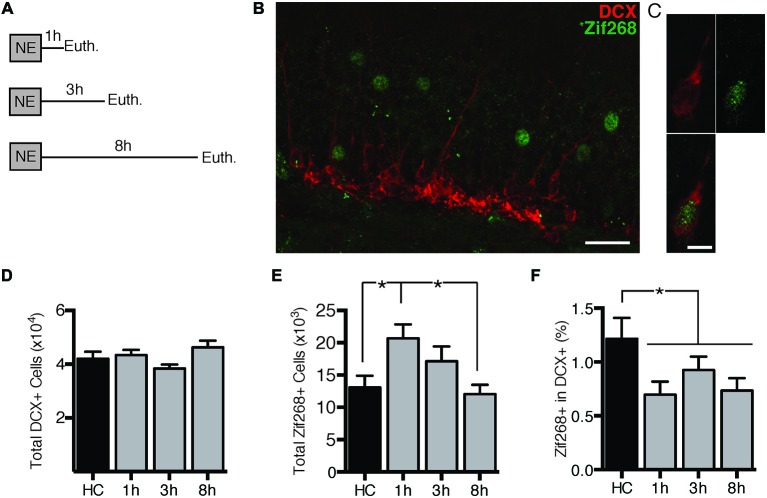
**Novel environment (NE) exposure activates zif268 expression in mature dentate granule cells (DGCs) but suppresses zif268 expression in immature DGCs. (A)** Mice were exposed to a novel environment for 10 min and euthanized 1 h (*n* = 7), 3 h (*n* = 7), or 8 h (*n* = 6) later. An additional group was euthanized immediately after being removed from the home cage (HC; *n* = 6). **(B,C)** Representative images of DCX and zif268 immunohistochemistry. Scale bars = 27 μm **(B)** and 9 μm **(C)**. **(D)** The total number of DCX+ cells did not differ among experimental conditions (*F*_(3,22)_ = 2.399, *p* = 0.095). **(E)** Total number of zif268+ cells in the granule cell layer (GCL) in HC and NE-exposed mice. NE exposure caused a significant increase in the number of DGCs expressing zif268 as compared to HC (*F*_(3,22)_ = 3.961, *p* = 0.021). Zif268 expression peaked 1 h after NE exposure. **(F)** Percentage of DCX+ cells expressing zif268 in HC and NE-exposed mice. The number of DCX+ cells expressing zif268 was significantly reduced as compared to the HC group (*t*_(24)_ = 2.520, *p* = 0.019) for at least 8 h after NE exposure. *p < 0.05.

First we assessed the time-course of zif268 expression in dentate granule cells (DGCs) after exposure to a novel environment (NE), a manipulation previously demonstrated to robustly induce IEG expression in DGCs (Clark et al., 2012). Mice were euthanized directly from the home cage (*n* = 6) or 1 (*n* = 7), 3 (*n* = 7), or 8 h (*n* = 6) after a 10-min exposure to a NE (Figure 1A). DCX expression was used to identify immature, adult-born neurons. We quantified overall zif268 expression in the general granule cell population as well as co-labeling of DCX with zif268 (Figures 1B,C). NE exposure caused a significant increase in the total number of zif268+ cells in the DG (*F*_(3,22)_ = 3.961, *p* = 0.021; Figure 1E). Pairwise comparisons revealed that the total number of zif268+ cells at 1 h post-exposure exceeded the home cage and 8 h levels (*p*’s ≤ 0.014); the other comparisons did not reach significance.

Next we examined expression of zif268 in DCX+ immature neurons. The home cage, 1, 3, and 8 h conditions had equivalent numbers of DCX+ neurons (*F*_(3,22)_ = 2.399, *p* = 0.095; Figure 1D). The percentage of DCX+ cells also expressing zif268 appeared to be reduced after NE exposure (Figure 1F). One-way ANOVA did not detect a significant effect across the four conditions (*F*_(3,22)_ = 2.241, *p* = 0.112). Because there were no significant differences among the NE-exposed groups (*p*’s ≥ 0.358), we conducted a second analysis with the NE data pooled across the 1, 3, and 8 h conditions. The percentage of DCX+ cells expressing zif268 was significantly higher in home cage mice than in mice exposed to the NE (*t*_(24)_ = 2.520, *p* = 0.019). In summary, exposure to a NE induced zif268 among the overall granule cell population but suppressed zif268 expression in DCX+ granule cells.

### Novel Environment Exposure, Enriched Environment Exposure and Water Maze Acquisition Produce Equivalent Zif268 Suppression in Immature Neurons

**Figure 2:**
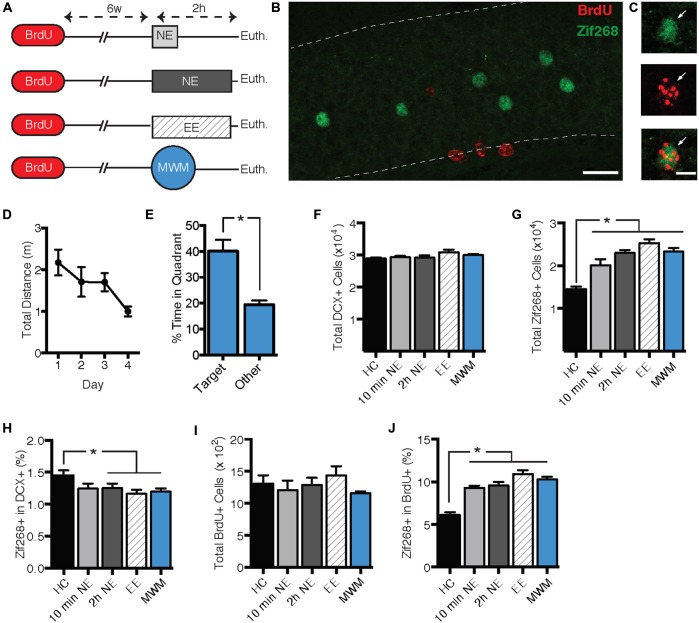
**Zif268 expression in DCX+ neurons was suppressed by exposure to a novel environment (NE), an enriched environment (EE), and morris water maze (MWM). (A)** Mice were injected with BrdU 6 weeks prior to being exposed to a NE for 10 min (*n* = 4) or 2 h (*n* = 6), EE for 2 h (*n* = 6), or MWM training (*n* = 8). An additional group was euthanized immediately after being removed from the home cage (HC; *n* = 6). **(B,C)** Representative images of BrdU and zif268 immunohistochemistry. Scale bars = 27 μm **(B)** and 9 μm **(C)**. **(D)** Performance in the MWM improved over the 4 days of training (*F*_(3,21)_ = 3.555, *p* = 0.032). **(E)** During a probe trial on the final day of training, mice displayed a significant preference for the target quadrant (*t*_(7)_ = 3.382, *p* = 0.012). **(F)** The number of DCX+ cells did not differ significantly across experimental conditions (*F*_(4,25)_ = 2.024, *p* = 0.122). **(G)** Total number of zif268+ cells in the GCL. As compared to the HC group, all of the behavioral manipulations caused a significant increase in the total number of DGCs expressing zif268 (*F*_(4,25)_ = 24.640, *p* < 0.001; *p*’s ≤ 0.001). **(H)** Percentage of DCX+ cells expressing zif268. Compared to the HC group, 2-h NE, EE, and MWM training significantly decreased the percentage of DCX+ cells expressing zif268 (*F*_(4,25)_ = 2.862; *p* = 0.044; *p*’s ≤ 0.048). **(I)** The number of BrdU+ cells did not differ among experimental conditions (*F*_(4.25)_ = 1.007, *p* = 0.422). **(J)** Percentage of BrdU+ cells expressing zif268. All of the behavioral manipulations significantly increased the percentage of BrdU+ cells expressing zif268 as compared to the HC group (*F*_(4,25)_ = 26.050, *p* < 0.0001;  *post hoc* *p*’s < 0.0001). *p < 0.05.

The second experiment had three objectives. First, we sought to replicate the zif268 suppression using a behavioral procedure that might produce a more robust effect on zif268 expression. Second, we sought to determine whether zif268 suppression can be induced by behavioral tasks other than NE exposure. Finally, we sought to determine whether the zif268 suppression effect is specific to immature adult-born neurons or is general to all adult-born neurons regardless of age. Mice were injected with BrdU 6 weeks prior to a 10-min NE exposure (*n* = 4), a 2-h NE exposure (*n* = 6), a 2-h enriched environment exposure (*n* = 6), or training in the Morris water maze (MWM; *n* = 8; Figure 2A). Mice were euthanized 2 h after the start of behavioral testing, except for the home cage condition (*n* = 6), in which mice were euthanized immediately after removal from the home cage. In the water maze condition, mice were euthanized on the fourth day of training. As shown in Figures 2D,E, maze performance improved over the 4 days of training (*F*_(3, 21)_ = 3.555, *p* = 0.032), and in a probe trial on the final day of training, mice displayed a significant preference for the target quadrant (*t*_(7)_ = 3.382, *p* = 0.012).

As shown in Figure 2G, all of the behavioral manipulations induced significant zif268 expression in the general granule cell population as compared to the home cage condition. ANOVA confirmed a significant main effect of behavioral condition (*F*_(4,25)_ = 24.640, *p* < 0.001). *Post hoc* comparisons confirmed that zif268 counts in each of the behavioral conditions exceeded those of the home cage condition (*p*’s ≤ 0.001). Next we examined zif268 expression in DCX+ immature granule cells. The number of DCX+ cells did not differ among behavioral conditions (*F*_(4,25)_ = 2.024, *p* = 0.122, Figure 2F), but the percentage of DCX+ cells co-expressing zif268 was reduced in the behavioral conditions as compared to the home cage control (*F*_(4,25)_ = 2.862, *p* = 0.044; Figure 2H). *Post hoc* pairwise comparisons indicated that the percentage of DCX+ cells expressing zif268 was reduced relative to home cage in the 2-hr NE, enriched environment, and water maze conditions (*p*’s ≤ 0.048).

Next we asked whether the zif268 suppression observed in immature adult-born neurons is maintained in mature adult-born cells. We assessed zif268 expression in 6-week-old BrdU+ cells in the GCL and SGZ (Figures 2B,C). The number of BrdU+ cells did not differ among the behavioral conditions (*F*_(4,25)_ = 1.007, *p* = 0.422, Figure 2I). In contrast to their effect in the DCX+ population, the behavioral manipulations increased the probability of zif268 expression in BrdU+ cells (Figure 2J) as compared to the home cage condition. ANOVA confirmed a significant effect of behavioral condition on the percentage of BrdU+ cells expressing zif268 (*F*_(4,25)_ = 26.050, *p* < 0.001), and *post hoc* tests indicated each of the four behavioral conditions exceeded home cage on this measure (*p*’s < 0.001).

In summary, exposure to NE, enriched environment, or water maze training evoked zif268 in mature granule cells but suppressed zif268 expression in DCX+ immature granule cells.

### Learning Suppresses Activity in Immature Adult-Born Granule Cells

**Figure 3:**
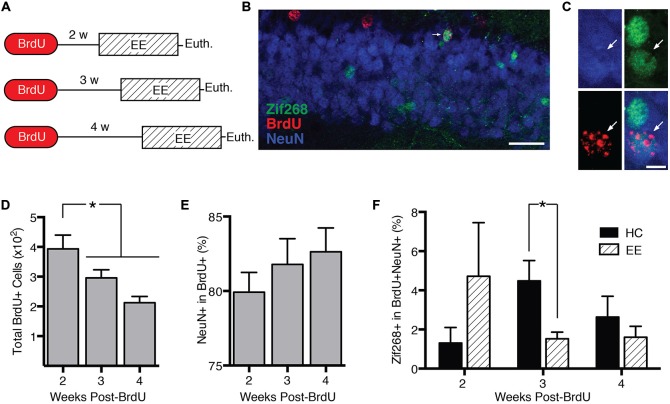
**Behavior-induced zif268 suppression is specific to 2–3-week-old adult-born neurons. (A)** We injected mice with BrdU 2 (*n* = 11), 3 (*n* = 12), or 4 weeks (*n* = 12) prior to euthanasia. Mice were euthanized 2 h after the start of enriched environment (EE) exposure (*n* = 17) or directly from the home cage (HC; *n* = 18). **(B,C)** Representative images of NeuN, BrdU, and zif268 immunohistochemistry. Scale bars = 27 μm **(B)** and 9 μm **(C)**. **(D)** Number of BrdU+ cells in the subgranular zone (SGZ) and granule cell layer (GCL) as a function of time after the BrdU injections. The number of BrdU+ cells declined over time (*F*_(2,32)_ = 7.665, *p* = 0.002; *p*’s ≤ 0.042). **(E)** Percentage of BrdU+ cells expressing NeuN. The percentage of BrdU+ cells expressing NeuN did not vary significantly over time after the BrdU injections (*F*_(2,32)_ = 0.755, *p* = 0.478). **(F)** Percentage of BrdU+ cells in SGZ and GCL expressing zif268. EE exposure significantly reduced the percentage of BrdU+ cells expressing zif268 relative to HC in the 3-week group (*t*_(10)_ = 2.696, *p* = 0.022) but not in the 2- or 4-week groups (*p*’s ≥ 0.225). *p < 0.05.

DCX+ cells range in age from 0 to approximately 4 weeks (Snyder et al., 2009a). In experiment 3, we characterized how behavior-induced zif268 expression varies across this age range. We injected mice with BrdU 2 (*n* = 11), 3 (*n* = 12), or 4 weeks (*n* = 12) prior to euthanasia (Figure 3A). Mice were euthanized 2 h after the start of enriched environment exposure (*n* = 17) or directly from the home cage (*n* = 18).

The number of BrdU+ cells declined over time after the BrdU injection (*F*_(2,32)_ = 7.665, *p* = 0.002; Figure 3D), consistent with other reports (Cameron et al., 1993). Pairwise comparisons confirmed that at 3 and 4 weeks post-injection, the number of BrdU+ cells had declined significantly below the 2-week level (*p*’s ≤ 0.042). Consistent with other reports (Snyder et al., 2009b; Stone et al., 2011), the percentage of BrdU+ cells exhibiting the neuronal marker NeuN did not vary over time after the BrdU injection (*F*_(2,32)_ = 0.755, *p* = 0.478; Figure 3E).

Next we examined how enriched environment-induced zif268 expression changed as a function of BrdU+ cell age (Figure 3F). We compared percentage of BrdU+/NeuN+ cells co-expressing zif268 in home cage vs. enriched environment groups as a function of week (Figures 3B,C). Enriched environment exposure significantly reduced zif268 expression relative to the home cage in 3-week-old BrdU+ neurons (*t*_(10)_ = 2.696, *p* = 0.022). There were no significant differences between home cage and enriched environment in either the 2- or 4-week-old BrdU+ cells (*p*’s ≥ 0.225). In summary, behavior-induced zif268 expression was limited to immature granule cells 2–3 weeks of age.

## Discussion

Our experiments yielded three main results: (1) Behavioral experience induces zif268 expression in mature granule cells but suppresses zif268 expression in immature adult-born granule cells; (2) zif268 suppression in immature granule cells is evoked by multiple types of behavioral experience; and (3) the time-course of behavior-induced changes in zif268 expression differs between young and mature granule cells, with behavior-induced zif268 suppression in immature neurons lasting longer than behavior-induced zif268 expression in mature granule neurons. The data confirm the presence of a transient cell-developmental period during which behavioral experience suppresses zif268 in immature granule cells.

Behavior-induced zif268 suppression did not appear to require a stressful behavioral experience as previously hypothesized (Snyder et al., 2012), because the magnitude of zif268 suppression was comparable across different tasks that would appear to be only mildly stressful. However, the possibility remains that the zif268 suppression would be stronger under more stressful conditions. Consistent with this idea, Snyder et al. (2012) observed that water maze-evoked zif268 expression in DCX+ cells was stronger in mice with previous water maze experience than in mice experiencing the maze for the first time. A definitive assessment of the role of stress in zif268 suppression will require assessing a variety of procedures that vary widely in the degree and type of stress they induce.

Behavior-induced zif268 suppression was observed in 3-week-old adult-born neurons but not in 2-, 4- or 6-week old neurons. Indeed, 6-week-old adult-born neurons exhibited robust induction of zif268 expression by behavioral experience, comparable to previous reports (Tashiro et al., 2007; Snyder et al., 2012; Jungenitz et al., 2014). We hypothesize that behavior-induced zif268 suppression in 3-week-old neurons relates to the developmental status of excitatory and inhibitory synapses in neurons of this age. At 2–3 weeks of age, the polarity of GABA responses transitions from depolarizing to hyperpolarizing, but depolarizing glutamatergic inputs are not yet mature (Espósito et al., 2005; Ge et al., 2006; Deshpande et al., 2013; Veyrac et al., 2013). Thus, activation of excitatory inputs into the DG may lead to predominantly hyperpolarizing responses in 3-week-old neurons. Because neuronal zif268 expression is activated by depolarizing inputs, hyperpolarization would be predicted to suppress zif268 expression. Alternatively, zif268 suppression could be attributable to developmental changes in expression of zif268 transcriptional repressors. For instance, zif268 can suppress its own expression through interactions with NAB1 and NAB2 (Russo et al., 1995; Svaren et al., 1996; Kumbrink et al., 2005). Upregulation of these repressors in immature neurons may be sufficient to produce behavior-induced zif268 suppression.

Behavior-induced suppression of zif268 appears to be unique among different IEGs. Expression of Arc, Homer1a, and cFos is very low or absent in neurons less than 3–4 weeks old (Jessberger and Kempermann, 2003; Kee et al., 2007; Snyder et al., 2009b; Stone et al., 2011), even after high-frequency electrical stimulation (Jungenitz et al., 2014). Consistent with these studies, we had difficulty identifying DCX+ cells co-expressing Arc or cFos (data not shown). Several studies also failed to detect zif268 in neurons less than 3 weeks old (Jessberger and Kempermann, 2003; Bruel-Jungerman et al., 2006; Snyder et al., 2009b; Jungenitz et al., 2014). However, these studies failed to include a home cage condition, which, in our hands produced higher levels of zif268 expression in immature neurons than did behavioral experience. Our results are consistent with those of Snyder et al. (2012), who found that zif268 expression in DCX+ cells was significantly stronger in home cage mice than in water maze-exposed mice. Our data indicate that resting zif268 expression can be detected in adult-born neurons as young as 2 weeks of age, but behavior-induced zif268 expression is not robust until after the DCX+ stage.

Zif268 suppression in immature granule cells could have two functional consequences. One consequence may be to prevent immature adult-born cells from being recruited into memory networks. In LTP studies, zif268 expression correlates not with the magnitude of LTP induction but rather with the persistence of LTP (Richardson et al., 1992; Abraham et al., 1993; Davis et al., 2003). Overexpression of zif268 in forebrain neurons enhances LTP in the DG (Penke et al., 2014), whereas knocking out zif268 leads to deficits in both the maintenance of late-LTP (Jones et al., 2001) and long-term memory (Katche et al., 2012). These deficits may result from a failure to activate the plasticity-related downstream targets of zif268 such as synapsin I/II, which are important in vesicle release and synaptogenesis (Thiel et al., 1994; Penke et al., 2014). Plasticity deficits could also be due to the disinhibition of proteasomal targets typically suppressed by zif268 activation, leading to degradation of plasticity-related proteins (James et al., 2005, 2006; McDade et al., 2009). Thus, suppression of zif268 in immature neurons may prevent these cells from being recruited into long-term memory networks. A second possibility is that behavior-induced zif268 suppression supports learning-induced death of immature neurons. Studies in rats indicate that participating in hippocampus-dependent learning tasks can promote survival (Gould et al., 1999; Shors et al., 2002) of adult-born neurons 1–2 weeks old at the start of training but induce death of neurons less than 7 days old at the start of training (Ambrogini et al., 2000, 2004; Döbrössy et al., 2003; Ehninger and Kempermann, 2006; Dupret et al., 2007). The pace of neuronal maturation is 1–2 weeks faster in rats than in mice (Snyder et al., 2009a); thus, learning-induced apoptosis in mice would be predicted to occur in cells 2–3 weeks of age, which corresponds to the period during which zif268 suppression was observed. Targets of zif268 include activity-induced neurotrophic factors such as BDNF, which are important in neuronal growth and survival (Baumgärtel et al., 2009). Because zif268 is required for activity-dependent survival of 2–3 weeks old neurons in mice (Veyrac et al., 2013), it is conceivable that behavior-induced zif268 suppression mediates learning-induced apoptosis of immature adult-born neurons.

In summary, our experiments identify a transient cell-developmental period during which behavioral experience suppresses zif268 expression in adult-born DGCs. Zif268 suppression occurred across three behavioral tasks differing in duration, stress level, and motivational basis; thus we conclude that zif268 suppression is a robust and general phenomenon present across a variety of conditions that activate excitatory inputs to the DG. We hypothesize that zif268 suppression prevents immature neurons from undergoing memory-related synaptic plasticity or mediates learning-induced apoptosis of immature adult-born neurons.

## Conflict of Interest Statement

The authors declare that the research was conducted in the absence of any commercial or financial relationships that could be construed as a potential conflict of interest.

## References

[B1] Abraham W. C. Mason S. E. Demmer J. Williams J. M. Richardson C. L. Tate W. P.. (1993). Correlations between immediate early gene induction and the persistence of long-term potentiation. Neuroscience 56, 717–727. 10.1016/0306-4522(93)90369-q 8255430

[B2] Altman J. Das G. D. (1965). Autoradiographic and histological evidence of postnatal hippocampal neurogenesis in rats. J. Comp. Neurol. 124, 319–335. 10.1002/cne.901240303 5861717

[B3] Ambrogini P. Cuppini R. Cuppini C. Ciaroni S. Cecchini T. Ferri P.. (2000). Spatial learning affects immature granule cell survival in adult rat dentate gyrus. Neurosci. Lett. 286, 21–24. 10.1016/s0304-3940(00)01074-0 10822143

[B4] Ambrogini P. Orsini L. Mancini C. Ferri P. Ciaroni S. Cuppini R. (2004). Learning may reduce neurogenesis in adult rat dentate gyrus. Neurosci. Lett. 359, 13–16. 10.1016/j.neulet.2003.12.123 15050700

[B5] Baumgärtel K. Tweedie-Cullen R. Y. Grossmann J. Gehrig P. Livingstone-Zatchej M. Mansuy I. M. (2009). Changes in the proteome after neuronal zif268 overexpression. J. Proteome Res. 8, 3298–3316. 10.1021/pr801000r 19374395

[B6] Bruel-Jungerman E. Davis S. Rampon C. Laroche S. (2006). Long-term potentiation enhances neurogenesis in the adult dentate gyrus. J. Neurosci. 26, 5888–5893. 10.1523/jneurosci.0782-06.2006 16738230 PMC6675234

[B7] Cameron H. A. McKay R. D. (2001). Adult neurogenesis produces a large pool of new granule cells in the dentate gyrus. J. Comp. Neurol. 435, 406–417. 10.1002/cne.1040 11406822

[B8] Cameron H. A. Woolley C. S. McEwen B. S. Gould E. (1993). Differentiation of newly born neurons and glia in the dentate gyrus of the adult rat. Neuroscience 56, 337–344. 10.1016/0306-4522(93)90335-d 8247264

[B9] Clark P. J. Bhattacharya T. K. Miller D. S. Kohman R. A. DeYoung E. K. Rhodes J. S. (2012). New neurons generated from running are broadly recruited into neuronal activation associated with three different hippocampus-involved tasks. Hippocampus 22, 1860–1867. 10.1002/hipo.22020 22467337 PMC3390440

[B10] Davis S. Bozon B. Laroche S. (2003). How necessary is the activation of the immediate early gene zif268 in synaptic plasticity and learning? Behav. Brain Res. 142, 17–30. 10.1016/s0166-4328(02)00421-7 12798262

[B11] Deshpande A. Bergami M. Ghanem A. Conzelmann K.-K. Lepier A. Götz M.. (2013). Retrograde monosynaptic tracing reveals the temporal evolution of inputs onto new neurons in the adult dentate gyrus and olfactory bulb. Proc. Natl. Acad. Sci. U S A 110, E1152–E1161. 10.1073/pnas.1218991110 23487772 PMC3607028

[B12] Döbrössy M. D. Drapeau E. Aurousseau C. Le Moal M. Piazza P.-V. Abrous D. N. (2003). Differential effects of learning on neurogenesis: learning increases or decreases the number of newly born cells depending on their birth date. Mol. Psychiatry 8, 974–982. 10.1038/sj.mp.4001419 14647395

[B13] Dupret D. Fabre A. Döbrössy M. D. Panatier A. Rodríguez J. J. Lamarque S.. (2007). Spatial learning depends on both the addition and removal of new hippocampal neurons. PLos Biol. 5:e214. 10.1371/journal.pbio.0050214 17683201 PMC1939885

[B14] Ehninger D. Kempermann G. (2006). Paradoxical effects of learning the Morris water maze on adult hippocampal neurogenesis in mice may be explained by a combination of stress and physical activity. Genes Brain Behav. 5, 29–39. 10.1111/j.1601-183x.2005.00129.x 16436186

[B15] Espósito M. S. Piatti V. C. Laplagne D. A. Morgenstern N. A. Ferrari C. C. Pitossi F. J.. (2005). Neuronal differentiation in the adult hippocampus recapitulates embryonic development. J. Neurosci. 25, 10074–10086. 10.1523/jneurosci.3114-05.2005 16267214 PMC6725804

[B16] Ge S. Goh E. L. K. Sailor K. A. Kitabatake Y. Ming G.-L. Song H. (2006). GABA regulates synaptic integration of newly generated neurons in the adult brain. Nature 439, 589–593. 10.1038/nature04404 16341203 PMC1420640

[B17] Gould E. Beylin A. Tanapat P. Reeves A. Shors T. J. (1999). Learning enhances adult neurogenesis in the hippocampal formation. Nat. Neurosci. 2, 260–265. 10.1038/6365 10195219

[B18] James A. B. Conway A. M. Morris B. J. (2005). Genomic profiling of the neuronal target genes of the plasticity-related transcription factor - Zif268. J. Neurochem. 95, 796–810. 10.1111/j.1471-4159.2005.03400.x 16248890

[B19] James A. B. Conway A. M. Morris B. J. (2006). Regulation of the neuronal proteasome by Zif268 (Egr1). J. Neurosci. 26, 1624–1634. 10.1523/jneurosci.4199-05.2006 16452686 PMC6675497

[B20] Jessberger S. Kempermann G. (2003). Adult-born hippocampal neurons mature into activity-dependent responsiveness. Eur. J. Neurosci. 18, 2707–2712. 10.1111/j.1460-9568.2003.02986.x 14656319

[B21] Jones M. W. Errington M. L. French P. J. Fine A. Bliss T. V. Garel S.. (2001). A requirement for the immediate early gene Zif268 in the expression of late LTP and long-term memories. Nat. Neurosci. 4, 289–296. 10.1038/85138 11224546

[B22] Jungenitz T. Radic T. Jedlicka P. Schwarzacher S. W. (2014). High-frequency stimulation induces gradual immediate early gene expression in maturing adult-generated hippocampal granule cells. Cereb. Cortex 24, 1845–1857. 10.1093/cercor/bht035 23425888

[B23] Katche C. Goldin A. Gonzalez C. Bekinschtein P. Medina J. H. (2012). Maintenance of long-term memory storage is dependent on late posttraining Egr-1 expression. Neurobiol. Learn. Mem. 98, 220–227. 10.1016/j.nlm.2012.08.001 22906840

[B24] Kee N. Teixeira C. M. Wang A. H. Frankland P. W. (2007). Preferential incorporation of adult-generated granule cells into spatial memory networks in the dentate gyrus. Nat. Neurosci. 10, 355–362. 10.1038/nn1847 17277773

[B25] Kumbrink J. Gerlinger M. Johnson J. P. (2005). Egr-1 induces the expression of its corepressor nab2 by activation of the nab2 promoter thereby establishing a negative feedback loop. J. Biol. Chem. 280, 42785–42793. 10.1074/jbc.m511079200 16260776

[B26] McDade D. M. Conway A.-M. James A. B. Morris B. J. (2009). Activity-dependent gene transcription as a long-term influence on receptor signalling. Biochem. Soc. Trans. 37, 1375–1377. 10.1042/bst0371375 19909279

[B27] Penke Z. Morice E. Veyrac A. Gros A. Chagneau C. LeBlanc P.. (2014). Zif268/Egr1 gain of function facilitates hippocampal synaptic plasticity and long-term spatial recognition memory. Philos. Trans. R. Soc. Lond. B Biol. Sci. 369:20130159. 10.1098/rstb.2013.0159 24298160 PMC3843890

[B28] Richardson C. L. Tate W. P. Mason S. E. Lawlor P. A. Dragunow M. Abraham W. C. (1992). Correlation between the induction of an immediate early gene,zif/268 and long-term potentiation in the dentate gyrus. Brain Res. 580, 147–154. 10.1016/0006-8993(92)90938-6 1504794

[B29] Russo M. W. Sevetson B. R. Milbrandt J. (1995). Identification of NAB1, a repressor of NGFI-A- and Krox20-mediated transcription. Proc. Natl. Acad. Sci. U S A 92, 6873–6877. 10.1073/pnas.92.15.6873 7624335 PMC41432

[B30] Shors T. J. Townsend D. A. Zhao M. Kozorovitskiy Y. Gould E. (2002). Neurogenesis may relate to some but not all types of hippocampal-dependent learning. Hippocampus 12, 578–584. 10.1002/hipo.10103 12440573 PMC3289536

[B31] Snyder J. S. Choe J. S. Clifford M. A. Jeurling S. I. Hurley P. Brown A.. (2009a). Adult-born hippocampal neurons are more numerous, faster maturing and more involved in behavior in rats than in mice. J. Neurosci. 29, 14484–14495. 10.1523/JNEUROSCI.1768-09.2009 19923282 PMC2830901

[B33] Snyder J. S. Glover L. R. Sanzone K. M. Kamhi J. F. Cameron H. A. (2009b). The effects of exercise and stress on the survival and maturation of adult-generated granule cells. Hippocampus 19, 898–906. 10.1002/hipo.20552 19156854 PMC2755652

[B32] Snyder J. S. Clifford M. A. Jeurling S. I. Cameron H. A. (2012). Complementary activation of hippocampal-cortical subregions and immature neurons following chronic training in single and multiple context versions of the water maze. Behav. Brain Res. 227, 330–339. 10.1016/j.bbr.2011.06.025 21736899 PMC3212609

[B34] Song J. Zhong C. Bonaguidi M. A. Sun G. J. Hsu D. Gu Y.. (2012). Neuronal circuitry mechanism regulating adult quiescent neural stem-cell fate decision. Nature 489, 150–154. 10.1038/nature11306 22842902 PMC3438284

[B35] Stone S. S. D. Teixeira C. M. Zaslavsky K. Wheeler A. L. Martinez-Canabal A. Wang A. H.. (2011). Functional convergence of developmentally and adult-generated granule cells in dentate gyrus circuits supporting hippocampus-dependent memory. Hippocampus 21, 1348–1362. 10.1002/hipo.20845 20824726

[B36] Svaren J. Sevetson B. R. Apel E. D. Zimonjic D. B. Popescu N. C. Milbrandt J. (1996). NAB2, a corepressor of NGFI-A (Egr-1) and Krox20, is induced by proliferative and differentiative stimuli. Mol. Cell. Biol. 16, 3545–3553. 8668170 10.1128/mcb.16.7.3545 PMC231349

[B37] Tashiro A. Makino H. Gage F. H. (2007). Experience-specific functional modification of the dentate gyrus through adult neurogenesis: a critical period during an immature stage. J. Neurosci. 27, 3252–3259. 10.1523/jneurosci.4941-06.2007 17376985 PMC6672473

[B38] Tashiro A. Sandler V. M. Toni N. Zhao C. Gage F. H. (2006). NMDA-receptor-mediated, cell-specific integration of new neurons in adult dentate gyrus. Nature 442, 929–933. 10.1038/nature05028 16906136

[B39] Thiel G. Schoch S. Petersohn D. (1994). Regulation of synapsin I gene expression by the zinc finger transcription factor zif268/egr-1. J. Biol. Chem. 269, 15294–15301. 8195167

[B40] Veyrac A. Gros A. Bruel-Jungerman E. Rochefort C. Kleine Borgmann F. B. Jessberger S.. (2013). Zif268/egr1 gene controls the selection, maturation and functional integration of adult hippocampal newborn neurons by learning. Proc. Natl. Acad. Sci. U S A 110, 7062–7067. 10.1073/pnas.1220558110 23569253 PMC3637756

